# Kinetic bed therapy to prevent nosocomial pneumonia in mechanically ventilated patients: a systematic review and meta-analysis

**DOI:** 10.1186/cc4912

**Published:** 2006-05-09

**Authors:** Anthony Delaney, Hilary Gray, Kevin B Laupland, Danny J Zuege

**Affiliations:** 1 Intensive Care Unit, Royal North Shore Hospital, Sydney, NSW, Australia; 2 Northern Clinical School, University of Sydney, St Leonards, NSW, Australia; 3 Department of Rehabilitation and Specialized Clinical Services, Calgary Health Region, Calgary, Alberta, Canada; 4 Department of Critical Care Medicine, University of Calgary, Calgary, Alberta, Canada; 5 Department of Community Health Sciences, University of Calgary, Calgary, Alberta, Canada; 6 Department of Medicine, University of Calgary, Calgary, Alberta, Canada

## Abstract

**Introduction:**

Nosocomial pneumonia is the most important infectious complication in patients admitted to intensive care units. Kinetic bed therapy may reduce the incidence of nosocomial pneumonia in mechanically ventilated patients. The objective of this study was to investigate whether kinetic bed therapy reduces the incidence of nosocomial pneumonia and improves outcomes in critically ill mechanically ventilated patients.

**Methods:**

We searched Medline, EMBASE, CINAHL, CENTRAL, and AMED for studies, as well as reviewed abstracts of conference proceedings, bibliographies of included studies and review articles and contacted the manufacturers of medical beds. Studies included were randomized or pseudo-randomized clinical trials of kinetic bed therapy compared to standard manual turning in critically ill mechanically ventilated adult patients. Two reviewers independently applied the study selection criteria and extracted data regarding study validity, type of bed used, intensity of kinetic therapy, and population under investigation. Outcomes assessed included the incidence of nosocomial pneumonia, mortality, duration of ventilation, and intensive care unit and hospital length of stay.

**Results:**

Fifteen prospective clinical trials were identified, which included a total of 1,169 participants. No trial met all the validity criteria. There was a significant reduction in the incidence of nosocomial pneumonia (pooled odds ratio (OR) 0.38, 95% confidence interval (CI) 0.28 to 0.53), but no reduction in mortality (pooled OR 0.96, 95%CI 0.66 to1.14), duration of mechanical ventilation (pooled standardized mean difference (SMD) -0.14 days, 95%CI, -0.29 to 0.02), duration of intensive care unit stay (pooled SMD -0.064 days, 95% CI, -0.21 to 0.086) or duration of hospital stay (pooled SMD 0.05 days, 95% CI -0.18 to 0.27).

**Conclusion:**

While kinetic bed therapy has been purported to reduce the incidence of nosocomial pneumonia in mechanically ventilated patients, the overall body of evidence is insufficient to support this conclusion. There appears to be a reduction in the incidence of nosocomial pneumonia, but no effect on mortality, duration of mechanical ventilation, or intensive care or hospital length of stay. Given the lack of consistent benefit and the poor methodological quality of the trials included in this analysis, definitive recommendations regarding the use of this therapy cannot be made at this time.

## Introduction

Nosocomial pneumonia is the most important infectious complication in patients admitted to intensive care units (ICUs), occurring in up to 50% of patients in high risk groups [1,2]. It has been associated with poor clinical and economic outcomes as well as an increased mortality risk in critically ill patients [1,3-5]. Nosocomial pneumonia associated with mechanical ventilation has been recognized as one of the most important preventable causes of morbidity and mortality in critically ill patients by the Institute of Healthcare Improvement [6]. The prevention of nosocomial pneumonia could significantly reduce morbidity, mortality and health care costs associated with critical illness.

One of the risk factors for nosocomial pneumonia in critically ill patients is prolonged immobilization associated with mechanical ventilation [7]. Patients who are nursed in a relatively static recumbent position have reduced muco-ciliary transport, atelectasis, and altered pulmonary venous flow [8,9]. It has been suggested that the use of kinetic beds in this patient group may overcome some of these physiological changes [8,10]. Kinetic bed therapy, which is known by a number of different names, including kinetic therapy, continuous lateral rotational therapy, oscillation therapy, and continuous postural oscillation, involves nursing the patient on a bed that continuously rotates in an attempt to prevent the respiratory complications of immobility.

Recent clinical practice guidelines for the prevention of ventilator associated pneumonia (VAP) have suggested that critical care providers should consider the use of kinetic bed therapy [11]. The true magnitude of effect of kinetic bed therapy on VAP remains unclear, however, and these recommendations may not have considered the collective effect of this therapy on more clinically important outcomes such as mortality, economic outcomes such as ICU or hospital length of stay, and the potential for important complications. Although a number of small studies have been reported over the recent decades, no single definitive trial has been conducted. A previous attempt at meta-analysis of this data was limited in that the authors focused only on one type of kinetic bed, did not include assessments of study quality, and did not use contemporary meta-analytic techniques [12]. As well, several additional studies have been published since presentation of this review.

To address these issues, we performed a systematic review and meta-analysis to investigate whether, for patients requiring mechanical ventilation in an intensive care unit, the use of kinetic bed therapy was associated with a lower incidence of nosocomial pneumonia compared to manual intermittent turning in a standard medical bed. We also sought to investigate the effect of this therapy on mortality, duration of mechanical ventilation, ICU length of stay and hospital length of stay and what complications were associated with the use of these beds.

## Materials and methods

### Search strategy

A number of sources were used to identify potentially relevant studies. The MEDLINE database was searched using the PubMed interface, and this search was supplemented by searches of the MEDLINE, EMBASE, CINAHL, AMED and Cochrane Central Register of Controlled Trials using the OVID interface. Search terms used were: ((rotat* and therapy) OR (rotat* and bed) OR (rotat* and lateral) or (oscillat* and bed) OR (oscillat* and therapy) or kinetic therapy or kinetic positioning or kinetic treatment table or continuous mechanical turning or continuous postural oscillation) combined with (pneumonia OR respiratory tract infection). There was no language restriction imposed on the search. There was no time limit imposed on the search, which was completed on 20 June, 2005. In addition, conference proceedings of the scientific meetings of the American Thoracic Society, American College of Chest Physicians, Society of Critical Care Medicine and European Society of Intensive Care Medicine from 2000 to 2004 were searched to identify unpublished studies. Bibliographies of review articles and included studies were reviewed and manufacturers of kinetic beds were contacted to identify otherwise unrecognized studies.

### Study selection

One author reviewed the titles and abstracts of all references to identify studies that could potentially meet the inclusion criteria. Two other authors independently applied the predetermined inclusion criteria to the potentially eligible studies to determine eligibility for inclusion, with disputes resolved by a third person. All published and unpublished studies were considered eligible if the available report contained sufficient information to assess the study for its potential eligibility. When sufficient information was not available in the report to determine study eligibility, validity or results, attempts were made to contact the authors to obtain this information. To be eligible the report had to describe a study: of critically ill adults receiving mechanical ventilation; where the intervention was a kinetic or rotating bed applied for at least 24 hours; where the control group received intermittent manual turns; which had a prospective randomized or pseudo-randomized design; and where the outcome measures included any of the incidence of nosocomial pneumonia, mortality, duration of mechanical ventilation, or ICU or hospital length of stay.

### Validity assessment

All included studies had their validity assessed independently by two authors, using standardized criteria, with disputes resolved by a third person. Each study was assessed in an unblinded fashion [13] and was evaluated for the adequacy of allocation concealment, the blinding of the diagnosis of pneumonia, the production of an intention to treat analysis and the presence of a predetermined definition of pneumonia. The clinical, laboratory and radiological features that were used to define pneumonia were also abstracted. Studies that used a method of randomization that did not maintain allocation concealment, such as alternate days or medical record numbers (that is to say. were pseudo-randomized) were recorded as not maintaining allocation concealment. When it was unclear or not stated in the report or abstract that the study had addressed these issues, it was recorded as absent [14].

### Data abstraction and study characteristics

Data abstraction was performed using specific data collection forms, independently, in duplicate by two authors, with any disagreements resolved by a third person. Data were extracted regarding the population under investigation, the type of bed used, the degree of rotation (defined as the complete arc of rotation), the frequency of rotation (how many times per hour the bed completed a full arc of rotation), number of hours per day the bed was rotated for, when the therapy commenced, how long it was continued for, and what percentage of study participants were mechanically ventilated. Data regarding potential complications of this therapy were collected when this was included in the report.

Outcome data were collected regarding the incidence of new cases of nosocomial pneumonia (as defined in each study), mortality (defined as the mortality for the longest period of follow-up; hospital mortality, as the most clinically important outcome, was preferred to ICU mortality when both were available), the duration of mechanical ventilation, and ICU and hospital length of stay. As it was not always possible to ascertain from the reports the timing of the diagnosis of pneumonia relative to the timing of the initiation and discontinuation of mechanical ventilation, we have termed reported pneumonia as 'nosocomial' rather than 'ventilator acquired'. When data were not available in the report or were in a form not amenable to inclusion in the meta-analysis, attempts were made to contact the lead author of the report to obtain additional information.

### Data synthesis

Agreement on the inclusion of studies was assessed using a kappa statistic. Statistical heterogeneity was assessed using the χ^2 ^statistic and the *I*^2 ^statistic with an *I*^2 ^> 50% indicating at least moderate heterogeneity [15]. When no significant heterogeneity was found, dichotomous outcomes were pooled using the method of Mantel and Haenszel to produce a pooled odds ratio (OR). Continuous outcomes were pooled using the standardized mean differences method of Cohen. Subgroup analysis was only attempted for the two major outcomes, the incidence of nosocomial pneumonia and mortality, due to the small numbers of studies in each subgroup for the other outcomes. To assess the potential effect of trial quality on the outcomes, studies that had adequate allocation concealment and studies that had adequate blinding were pooled separately. As some authors have suggested that the degree of rotation may be important for the prevention of pneumonia [8], we performed subgroup analysis for those beds that rotated less than 80 degrees and those that rotated 80 degrees or more. The potential for publication bias was assessed using funnel plots and the statistical test described by Egger [16]. All analyses were conducted using STATA 8.2 (Statacorp, College Station, Texas, USA).

## Results

### Search results

**Figure 1:**
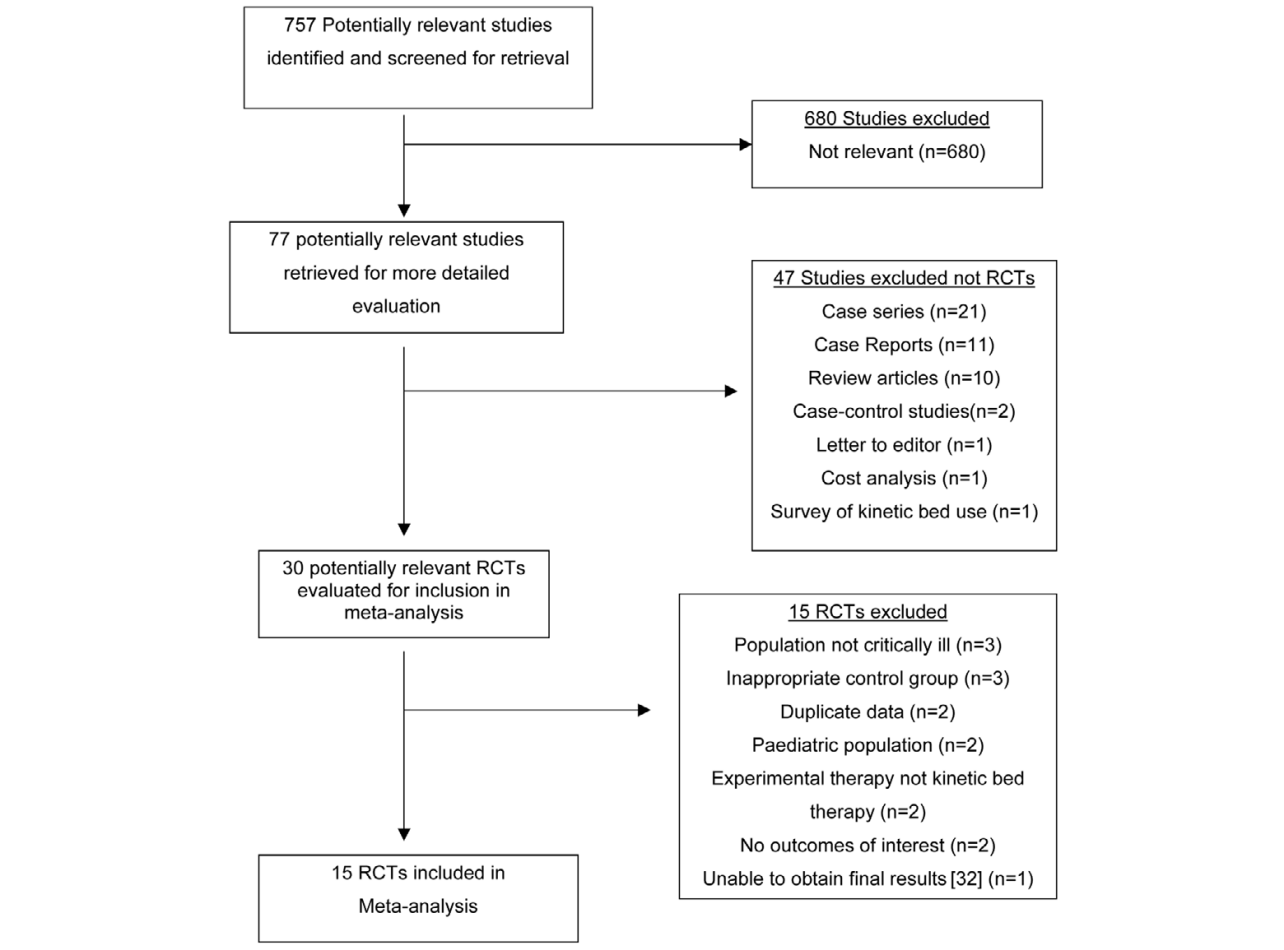
QUOROM Flow diagram summarizing trial flow and reasons for exclusion of studies. RCT, randomized clinical trial.

A total of 757 potentially relevant studies were identified. Fifteen studies were deemed eligible for inclusion in the meta-analysis [7,17-30]. One study that was included in a previous meta-analysis [12] was only available as an unpublished abstract reporting preliminary data [31]. The complete data could not be obtained despite multiple attempts to contact the authors and sponsor of this study; hence the study was excluded. Agreement on the inclusion of studies was reached in 96.1% of cases, giving a kappa value of 0.87. The flow of studies and reasons for exclusion of studies is shown in Figure 1. Significant clinical heterogeneity in the populations under investigation, the type of bed used, the degree of rotation, and the frequency and duration of therapy was evident, as shown in Table 1. In all 15 studies a total of 1,169 participants were randomized, the largest study had a total sample size of 255 participants. The methodological quality of the studies was generally poor as shown in Table 2. No study fulfilled all of the validity criteria. The results of each individual study are displayed in Table 3, with the complications noted in the intervention and control groups in each study shown in Table 4.

### Effect of kinetic bed therapy on the incidence of nosocomial pneumonia

**Figure 2:**
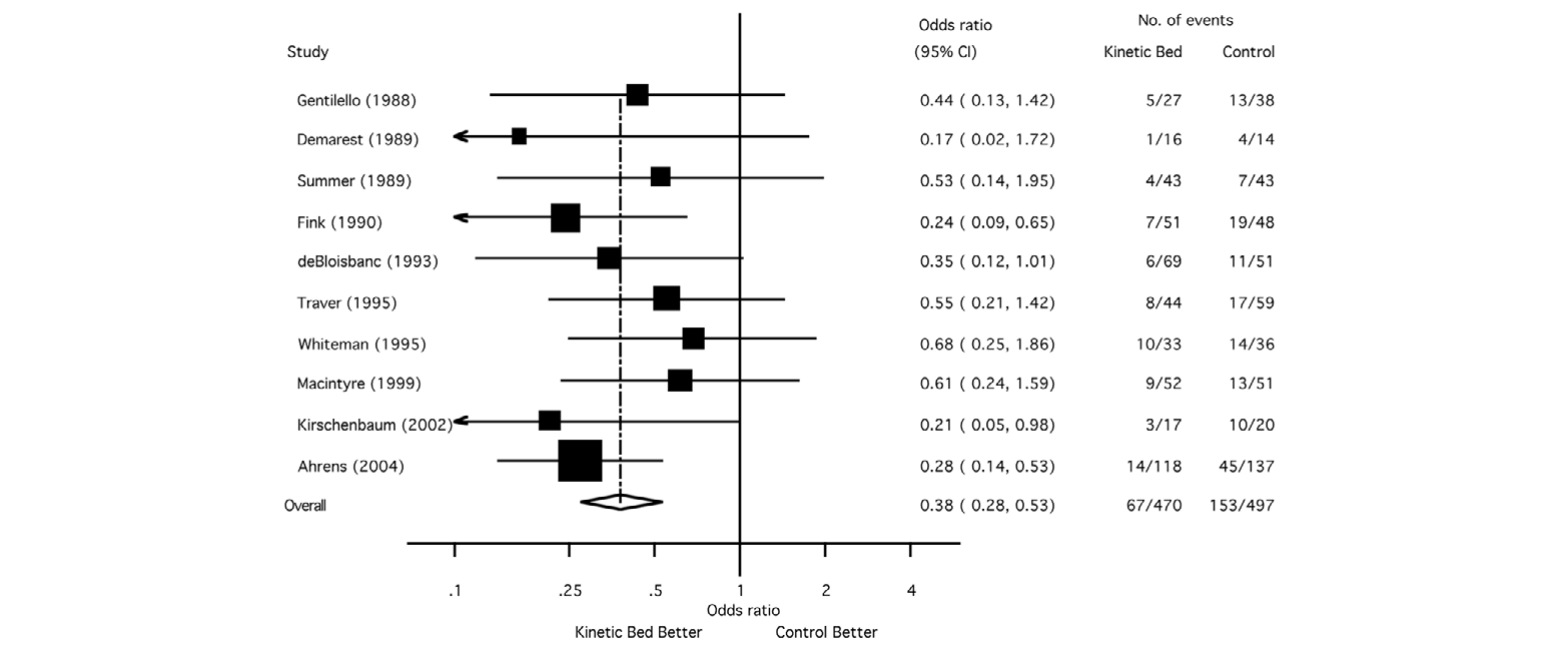
Forest plot showing the effect of kinetic bed therapy on nosocomial pneumonia

The incidence of pneumonia was reported in 10 studies [7,17,19-23,26-28]. There were five studies that reported the incidence of pneumonia that had adequate allocation concealment and six studies that had adequate blinding of outcome adjudication. There was no evidence of publication bias on inspection of the funnel plot (Additional file 1) or by Egger's bias statistic (bias = -0.12, *p *= 0.91). There was no statistical evidence of overall heterogeneity (χ^2 ^*p *= 0.64, *I*^2 ^= 0%). The pooled estimate from all 10 studies (Figure 2) revealed an estimated OR of 0.38 (95% confidence interval (CI) 0.28 to 0.53, *p *< 0.001), indicating a significant reduction in the odds of developing nosocomial pneumonia in patients treated with kinetic bed therapy. This reduction was consistent in studies with adequate allocation concealment (OR = 0.38, 95%CI 0.23 to 0.62) and studies without adequate allocation concealment (OR = 0.39, 95%CI 0.25 to 0.59) with the test for heterogeneity between subgroups *p *= 0.94 (Additional file 2). However, there was a trend for studies without blinding of outcome adjudication (OR = 0.28, 95%CI 0.17 to 0.46) to show a greater effect than studies with blinding of outcome adjudication (OR = 0.50, 95%CI 0.32 to 0.77) with the test for heterogeneity between subgroups *p *= 0.09 (Additional file 3). When the results of studies that reported the arc of rotation were pooled, the estimate of the OR for the effect of kinetic bed therapy on the incidence of pneumonia was similar in studies with an arc of rotation of less than 80° (OR = 0.49, 95%CI 0.27 to 0.90) and those with an arc of rotation of 80° or more (OR = 0.36, 95% CI 0.24 to 0.52), with the test for heterogeneity between subgroups being non-significant (*p *= 0.53) (Additional file 4).

### Effect of kinetic bed therapy on mortality

**Figure 3:**
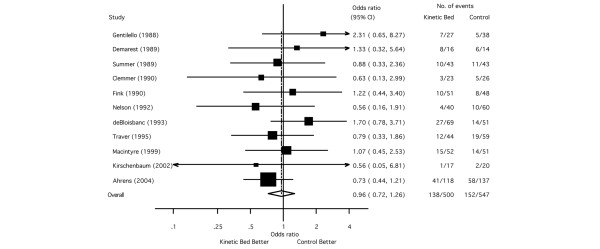
Forest plot showing the effect of kinetic bed therapy on mortality. CI, confidence interval.

Eleven studies reported the effect of kinetic bed therapy on mortality [7,17-24,26,27] (Figure 3). There were five studies with adequate allocation concealment. Again, there was no evidence of publication bias on inspection of a funnel plot (Additional file 5) or by Eggers statistic (bias = 0.33, *p *= 0.65). There was no evidence of overall statistical heterogeneity (χ^2 ^*p *= 0.73, *I*^2 ^= 0%). The pooled estimate of the OR for mortality with the use of kinetic bed therapy was 0.96 (95% CI 0.72 to 1.26, *p *= 0.75), indicating no significant reduction. This estimate of treatment effect was different in studies with adequate allocation concealment (OR 1.44, 95%CI 0.89 to 2.36) versus those without adequate allocation concealment (OR 0.78, 95%CI 0.55 to 1.10) with a test of heterogeneity between subgroups *p *= 0.045 (Additional file 6). The estimate of treatment effect on mortality was similar in studies using an arc of less than 80° (OR = 0.89, 95%CI 0.50 to 1.61) and those using an arc of 80° or more (OR 1.0, 95%CI 0.72 to 1.41), with the test of heterogeneity between subgroups *p *= 0.75 (Additional file 7).

### Effect of kinetic bed therapy on duration of mechanical ventilation

**Figure 4:**
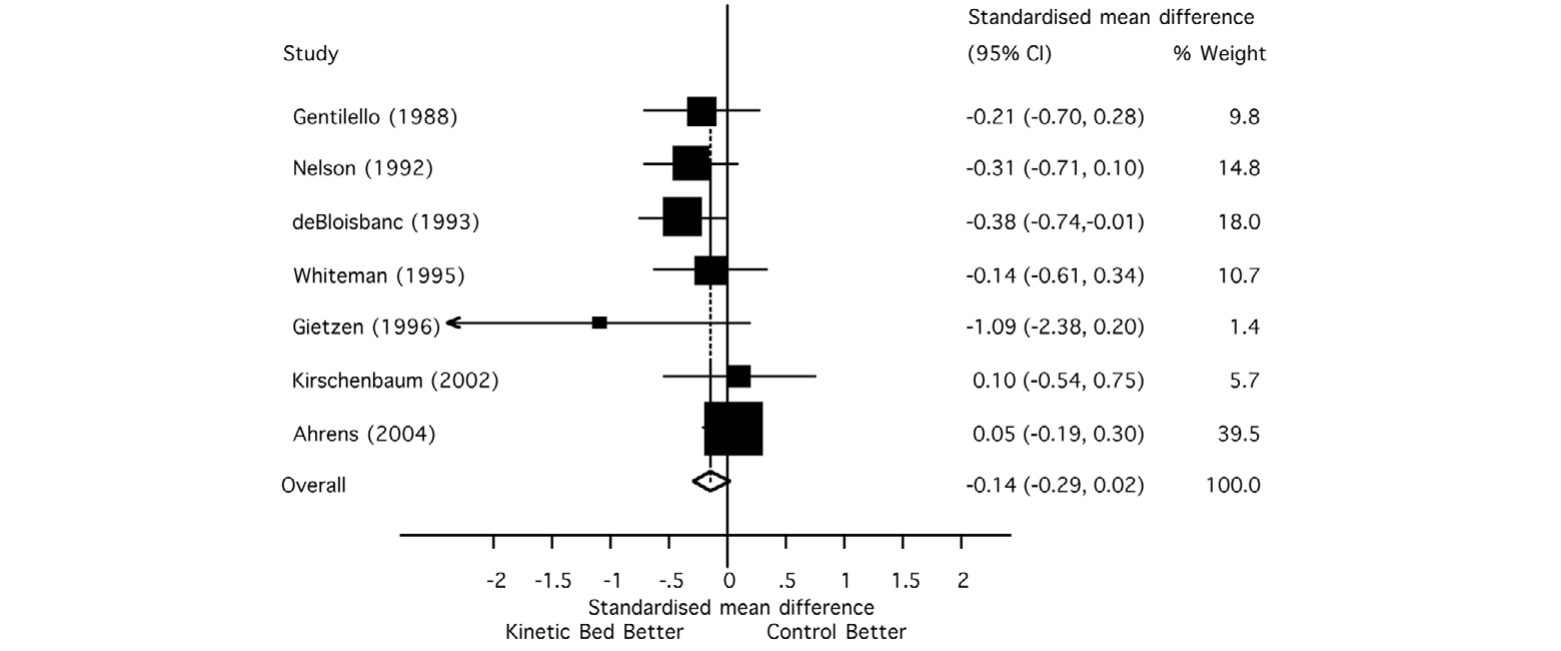
Forest plot showing the effect of kinetic bed therapy on duration of mechanical ventilation. CI, confidence interval.

Two studies [21,27] reported the duration of mechanical ventilation, ICU length of stay and hospital length of stay only as medians and range. The original data were not available to transform these results into means and standard deviations that would allow inclusion in the meta-analysis. Seven studies [17,19,22-24,28,30] reported on the effect of kinetic bed therapy on the duration of mechanicalventilation with the results reported in a way that allowed inclusion in a meta-analysis (Figure 4). There was no evidence of significant statistical heterogeneity (χ^2 ^= 7.34, *p *= 0.29 and *I*^2 ^= 18.3%). There was no discernable effect of kinetic bed therapy on the duration of mechanical ventilation (pooled standardized mean differences (SMD) = -0.14 days, 95%CI -0.29 to 0.02, *p *= 0.08).

### Effect of kinetic bed therapy on duration of ICU stay

**Figure 5:**
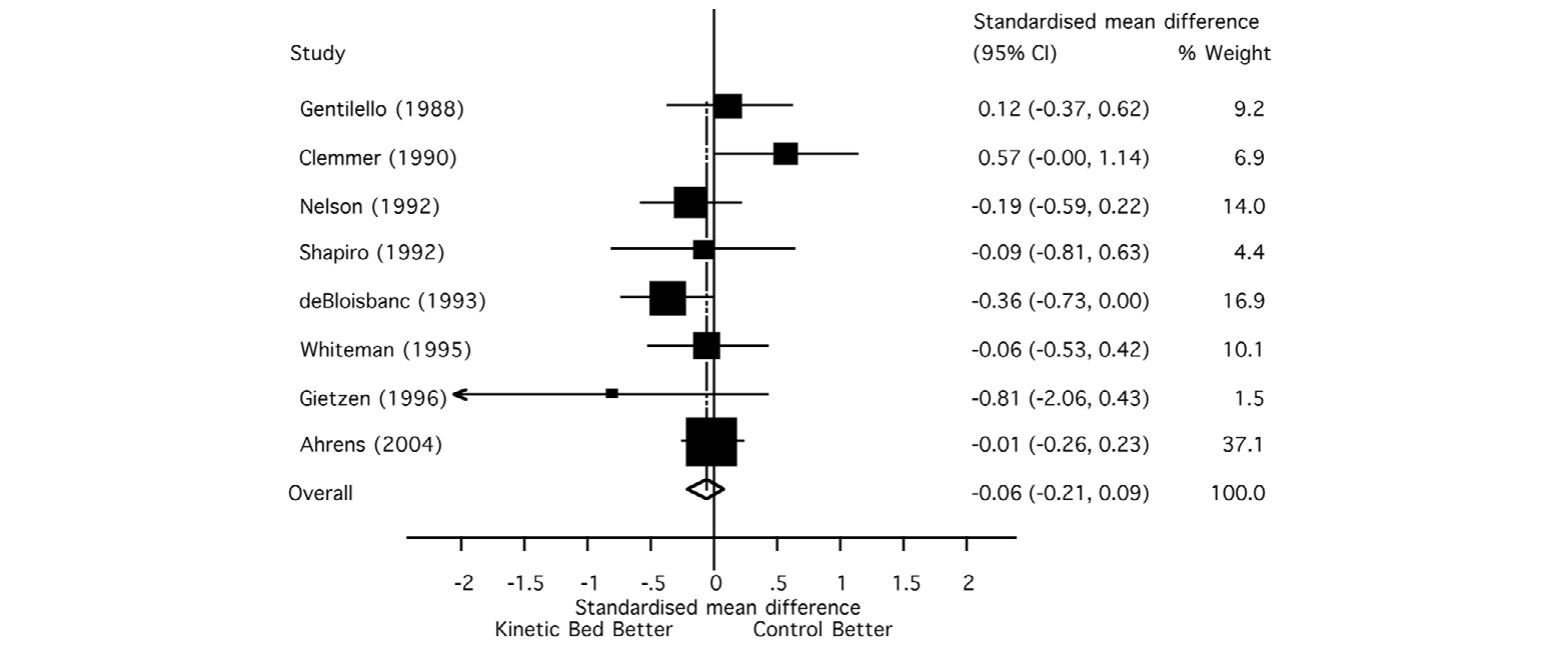
Forest plot showing the effect of kinetic bed therapy on intensive care unit length of stay. CI, confidence interval.

Eight studies [17-19,22,24,25,28,30] examined the effect of kinetic bed therapy on the duration of ICU stay (Figure 5); no significant statistical heterogeneity was apparent (χ^2 ^= 9.72, *p *= 0.21 and *I*^2 ^= 28%). Kinetic bed therapy had no discernable effect on the duration of ICU length of stay (pooled SMD = -0.064 days, 95%CI -0.21 to 0.086, *p *= 0.40).

### Effect of kinetic bed therapy on duration of hospital stay

**Figure 6:**
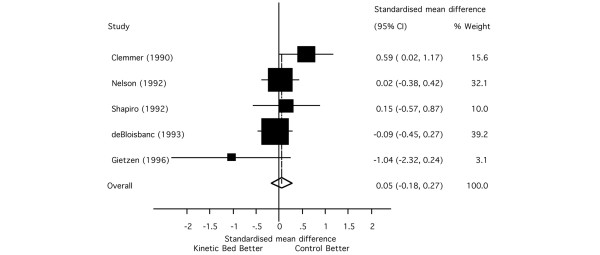
Forest plot showing the effect of kinetic bed therapy on hospital length of stay. CI, confidence interval.

A total of five studies [18,19,24,25,30] examined the effect of kinetic bed therapy on the length of hospital stay (Figure 6). There was no significant statistical heterogeneity (χ^2 ^= 6.91, *p *= 0.14 and *I*^2 ^= 42.1%). There was no apparent effect of kinetic bed therapy on the duration of hospital stay (pooled SMD = 0.05 days, 95%CI -0.18 to 0.27, *p *= 0.69).

## Discussion

The development of nosocomial pneumonia in patients admitted to the ICU has been recognized as one of the most important contemporary safety issues in critical care medicine. The 100 K Lives Campaign of the Institute for Health Care Improvement has identified the prevention of ventilator associated nosocomial pneumonia as a priority area. This study is important because it systematically examines the evidence that kinetic bed therapy reduces the incidence of nosocomial pneumonia in mechanically ventilated patients, the most important acquired infectious complication affecting the critically ill. Although guidelines for prevention have suggested the use of kinetic bed therapy based on systematic review, this study formally quantifies the magnitude in reduction in the incidence of nosocomial pneumonia that may be expected with the use of this therapy. However, given the uncertainty regarding the quality of the studies that have examined this issue, and the inconsistent effects on mortality, duration of mechanical ventilation, ICU and hospital length of stay, it would be premature to recommend widespread adoption of this therapy without further methodologically sound trials that considered all important potential benefits and adverse effects.

Although this study found a significant reduction in the odds of developing pneumonia in mechanically ventilated adults who received kinetic bed therapy, the pooled estimate reported should be interpreted carefully. Given that ventilator associated nosocomial pneumonia is known to be associated with a longer duration of mechanical ventilation and ICU length of stay [3], it may be expected that a reduction in the incidence of nosocomial pneumonia should result in a significant reduction in these other important outcomes. However, the reduction in pneumonia found in the pooled results in this meta-analysis was not associated with a significant reduction in duration of ventilation, ICU or hospital length of stay. One possible explanation could be that as secondary outcomes the power of the studies to identify smaller but clinically significant reductions in these outcomes was inadequate. It is also possible that the reduction in the number of patients diagnosed with nosocomial pneumonia was artefactual due to methodological deficiencies in the included clinical trials, rather than a true reduction. This possibility is supported by our observation that none of the trials included in this review fulfilled all of the validity criteria.

As the diagnosis of pneumonia is a more subjective outcome than mortality, duration of mechanical ventilation, or ICU length of stay, it may be more vulnerable to bias, and this may in part explain the marked reduction in pneumonia found in these studies. In particular, the exaggerated estimate of treatment effect in studies without adequate blinding would support this contention. Lack of allocation concealment is known to be associated with significant bias in the results of randomized controlled trials [14]. In this meta-analysis, studies that did not have adequate concealment of allocation showed a trend to a reduction in mortality, while those with adequate methods of allocation concealment showed a non-significant trend to an increase in mortality with kinetic bed therapy. The fact that many of the trials excluded patients who were unable to tolerate kinetic bed therapy means many of these analyses were not conducted using the principle of intention to treat. When trials that have major methodological flaws such as these are pooled, the results need to be interpreted with caution. Although the true effect of kinetic bed therapy on the incidence of nosocomial pneumonia may be overestimated in our analysis, it may still be of a clinically significant magnitude.

Another possible reason why a reduction in the incidence of pneumonia would not be associated with improvements in other outcomes would be if the therapy was associated with other complications that adversely affected these outcomes. That the potential complications of this therapy have not been systematically addressed in most of the studies is of serious concern. Of particular note is the study by MacIntyre and colleagues [7], in which there was an increased rate of cardiac arrests, unplanned extubations and new arrhythmias in the patients who were treated with kinetic bed therapy. These types of complications were not assessed in most of the other studies. The potential impact of the need for increased sedation also needs to be assessed to fully evaluate the effects of this therapy. It is possible that these complications may be associated with a morbidity and mortality that negates any benefit derived from the prevention of nosocomial pneumonia. Without a more thorough evaluation of these potential complications, it is difficult to make strong recommendations regarding the use of this therapy.

A number of other therapies have been documented to be useful for the prevention of pneumonia in ventilated patients and these have formed the basis of recent clinical practice guidelines [11,32]. These strategies include elevation of the head of the bed, use of endotracheal tubes allowing continuous aspiration of subglottic secretions, and sedation and ventilator weaning strategies that allow for extubation as rapidly as possible [11,32]. However, these simple, relatively inexpensive and effective therapies are yet to universally implemented [33]. Whether kinetic bed therapy would be as effective in preventing pneumonia if these other simple therapies were universally implemented is not clear.

There are a number of limitations to this review. Firstly, as has already been mentioned, the methodological quality of most of the included studies is poor. It is possible that our search did not identify all randomized clinical trials that have examined this issue, although our extensive search using multiple databases, use of multiple search terms, and contact with industry make this unlikely. We expect the effect of publication bias in this meta-analysis to be minimal given that we found no evidence of publication bias on examination of funnel plots, and we made extensive attempts to find unpublished material by contact with all known manufacturers of kinetic beds in North America. It is possible that a more marked effect of this therapy is possible in particular subgroups of patients, such as trauma patients or those more severely ill. As we were not able to obtain individual data for these patients these issues will need to be addressed by other means.

A number of important questions remain unanswered. The first and most important is whether the apparent reduction in nosocomial pneumonia that is associated with kinetic bed therapy can be reproduced in a rigorously conducted, adequately powered, clinical trial [34]. Whether this reduction in pneumonia is associated with improvements in other important outcomes such as mortality, duration of mechanical ventilation, and ICU and hospital length of stay, remains to be seen. Without a properly designed trial, determining whether the apparent benefits of this therapy are worth the risks and costs is very difficult. Such a study must include a full evaluation of the potential harms that could arise from the use of this therapy. With this information, a cost-utility analysis could be performed to help guide physicians and health care administrators in deciding the place of this therapy in preventing nosocomial pneumonia in mechanically ventilated patients.

## Conclusion

We conclude that kinetic bed therapy is associated with a significant reduction in the odds of developing nosocomial pneumonia in mechanically ventilated patients. However, it is not associated with a significant reduction in the mortality, duration of mechanical ventilation, or ICU or hospital length of stay. Given the lack of consistent benefit and the poor methodological quality of the clinical trials included in this analysis, definitive recommendations regarding the use of this therapy cannot be made at this time.

## Key messages

• Numerous studies have examined the utility of kinetic bed therapy to prevent nosocomial pneumonia in mechanically ventilated patients, but the methodological quality of these studies is not generally of a high standard.

• Kinetic bed therapy is associated with a reduction in the odds of developing nosocomial pneumonia compared to standard intermittent manual turning.

• The use of kinetic bed therapy is not associated with a reduction in mortality, duration of mechanical ventilation or duration of ICU or hospital length of stay.

• Potential complications of kinetic therapy have rarely been systematically reported in the clinical trials conducted to date.

• Until the results of further high quality clinical trials are available, the routine use of kinetic bed therapy to prevent nosocomial pneumonia in mechanically ventilated patients is not recommended.

## Abbreviations

CI = confidence interval; ICU = intensive care unit; OR = odds ratio; SMD = standardized mean differences; VAP = ventilator associated pneumonia.

## Competing interests

The authors declare that they have no competing interests.

## Authors' contributions

AD developed the study protocol, conducted the initial search for studies, assisted in study selection and data extraction, analyzed the data, and wrote and revised the manuscript. HG developed the study protocol, assisted in the search and assisted in the writing and revision of the manuscript. KL conceived the study, developed the study protocol, selected studies and extracted data, and assisted in writing and revising the manuscript. DZ conceived the study, developed the study protocol, contacted manufacturers and authors of the RCTs, selected studies and extracted data, and assisted in the writing and revision of the manuscript. All authors read and approved the final manuscript.

## Supplementary Material

Additional file 1 Click here for file

Additional file 2 Click here for file

Additional file 3 Click here for file

Additional file 4 Click here for file

Additional file 5 Click here for file

Additional file 6 Click here for file

Additional file 7 Click here for file

## Figures and Tables

**Table 1:** Summary of the characteristics of randomized clinical trials of kinetic bed therapy

Study	Type of bed	Rotation	Frequency	Hours per day	Therapy commenced	Duration of therapy	Population	Severity of illness score (treatment v control)	Ventilated
Ahrens [17]	Trial Dyne II KCI	80°	2 per hour	18	NR	NR	Medical, surgical, trauma	APACHE II 27.2 v 27.3	100%
Bhazad [29]	Rotobed, KCI	120°	30 per hour	NR	NR	3 days	ARDS	NR	100%
Kirschenbaum [23]	EFICA Hill-Rom	60°	NR	18	Day 1	NR	Chronic ventilation Unit	APACHE II 16.9 v 16.9	100%
Macintyre [7]	Restcue Support Systems International	64°	8 per hour	24	NR	ICU discharge	General medical/surgical	APACHE II 16.7 v 16.4	100%
Gietzen [30]	Biodyne KCI	80°	3 per hour	NR	NR	ICU discharge	ARDS	APACHE II 14.6 v 15.3	100%
Traver [27]	Biodyne KCI	51°	2 per hour	NR	Day 1	ICU discharge or mobile	General medical/surgical	APACHE II 19.8 v 18.2	89%
Whiteman [28]	Restcue Support Systems International	120°	8 per hour	NR	Day 2	ICU discharge or mobile	Pre and post liver transplant	APACHE II 21.2 v 23.6	100%
deBloisbanc [19]	Biodyne KCI	90°	8.5 per hour	18	Day 1	5 days	Medical	APACHE II 18.5 v 16.8	79%
Nelson [24]	Kinetic Treatment Table KCI	NR	NR	16	Day 1	Until patient was mobile	Trauma	ISS 32.9 v 33.0	100%
Shapiro [25]	Kinetic Treatment Table KCI	84° to124°	NR	14	NR	Until patient was mobile	Trauma	ISS 45 v 29	>75%
Clemmer [18]	Kinetic Treatment Table KCI	144°	NR	17	Day 1–2	8 days	Traumatic brain injury	ISS 42.9 v 40.8	100%
Fink [21]	Kinetic Treatment Table KCI	80°	NR	10 to 16	Day 1	ICU discharge	Trauma	ISS 34 v 35	96%
Demarest [20]	Kinetic Treatment Table KCI	NR	NR	12.2	Average 1.9 days	7 days	Trauma	ISS 29.4 v 27.9	100%
Summer [26]	Kinetic Treatment Table KCI	120°	17 per hour	NR	Day 1	Until alert	Medical	APACHE II 17 v 19	100%
Gentilello [22]	Kinetic Treatment Table KCI	124°	8.5 per hour	13.4	Day 1	ICU discharge	Trauma	Trauma score 12.0 v 11.5	100%

APACHE, Acute Physiology and Chronic Health Evaluation; ARDS, acute respiratory distress syndrome; ICU, intensive care unit; ISS, injury severity score;  KCI, Kinetic Concepts Inc. NR, not reported.

**Table 2:** Summary of the validity assessments of the randomized clinical trials of kinetic bed therapy

Study	Year	Allocation concealment	Blinding	Intention-to-treat analysis	Explicit definition of pneumonia	Features used to define of pneumonia
Ahrens [17]	2004	No	No	No	Yes	C, F, L, SA, H, BC, PC
Bhazad [29]	2002	Yes	No	No	No	Nil
Kirschenbaum [23]	2002	No	Yes	No	Yes	C, F, SC, BQ
Macintyre [7]	1999	No	Yes	No	Yes	C, F, L, SA, BQ
Gietzen [30]	1996	No	No	No	No	Nil
Traver [27]	1995	No	Yes	No	Yes	C, F, L
Whiteman [28]	1995	Yes	Yes	No	Yes	C, F, L, SA, SC
deBloisbanc [19]	1993	Yes	Yes	No	Yes	C, F, SA
Nelson [24]	1992	No	No	No	Yes	C, F, L, SA
Shapiro [25]	1992	No	No	No	No	Nil
Clemmer [18]	1990	Yes	Yes	No	No	Nil
Fink [21]	1990	Yes	No	Yes	Yes	C, F, SA, SC
Demarest [20]	1989	Yes	No	No	Yes	C, F, SA
Summer [26]	1989	No	Yes	No	Yes	C, F, L, SA
Gentilello [22]	1988	Yes	No	No	Yes	C, F, L, SA, SC

BC, blood culture; BQ, bronchoalveolar lavage with quantitative culture; C, persistent new changes on chest radiograph; F, fever; H, histologic evidence of pneumonia in lung tissue; L, leukocytosis or leukopaenia; PC, pleural fluid culture; SA, altered sputum appearance; SC, positive sputum culture.

**Table 3:** Summary of the results for each of the randomized clinical trials of kinetic bed therapy

Study	Sample size	Nosocomial pneumonia (treatment vs control)	Mortality (treatment vs control)	Ventilated days (treatment vs control)	ICU LOS (treatment vs control)	Hospital LOS (treatment vs control)
Ahrens [17]	255	14/118 vs 45/137	41/118 vs 58/137	10.8 ± 12.2 vs 10.1 ± 10.6	13.5 ± 13.2 vs 13.6 ± 11.3	NR
Bhazad [30]	22	NR	NR	NR	10 vs 18^a^	NR
Kirschenbaum [23]	37	3/17 vs 10/20	1/17 vs 2/20	21.0 ± 9.9 vs 20.0 ± 9.4	NR	NR
Macintyre [7]	103	9/52 vs 13/51	15/52 vs 14/51	NR	NR	NR
Gietzen [30]	11	NR	NR	12.4 ± 3.8 vs 35.5 ± 31.5	17.2 ± 5.8 vs 24.3 ± 11.4	25.0 ± 10.9 vs 44.8 ± 25.8
Traver [27]	103	8/44 vs 17/59	12/44 vs 19/59	3.0 (0–28) vs 3.0 (0–24)^b^	7.0 (2–43) vs 5.0 (2–53)^b^	17.5 (3–98) vs 17.0 (3–74)^b^
Whiteman [29]	69	10/33 vs 14/36	NR	13.8 ± 11.5 vs 16.1 ± 20.6	29.8 ± 27.5 vs 32.0 ± 46.5	NR
deBloisbanc [19]	110	6/69 vs 11/51	27/69 vs 14/51	6.1 ± 7.5 vs 9.9 ± 12.9	7.8 ± 6.7 vs 10.8 ± 10.0	17.0 ± 18.3 vs 18.5 ± 13.6
Nelson [24]	100	NR	4/40 vs 10/60	6.9 ± 8.9 vs 10.9 ± 15.2	8.6 ± 11.7 vs 11.2 ± 15.4	32.9 ± 30.2 vs 32.2 ± 28.2
Shapiro [25]	30	NR	NR	NR	11.4 ± 10.4 vs 12.3 ± 10.1	25.6 ± 20.8 vs 22.6 ± 19.5
Clemmer [18]	49	NR	3/23 vs 5/26	NR	20.9 ± 16.2 vs 13.9 ± 7.5	27.1 ± 16.5 vs 19.0 ± 10.5
Fink [21]	99	7/51 vs 19/48	10/51 vs 8/48	4 (0–32) vs 7 (0–74)^b^	5 (1–32) vs 8 (2–74)^b^	20 (2–201) vs 37 (5–612)^b^
Demarest [20]	30	1/16 vs 4/14	8/16 vs 6/14	NR	15.1 vs 11.4^a^	NR
Summer [26]	86	4/43 vs 7/43	10/43 vs 11/43	NR	6.7 vs 11.6^a^	NR
Gentilello [22]	65	5/27 vs 13/38	7/27 vs 5/38	8.5 ± 5.3 vs 10 ± 8.2	16.8 ± 13.6 vs 15.0 ± 15.6	NR

^a^Data reported as mean only. ^b^Data reported as median and range. ICU, intensive care unit; LOS, length of stay; NR, not reported.

**Table 4:** Summary of the complications noted in randomized clinical trials of kinetic bed therapy

Study	Complications	
	Kinetic bed therapy group	Control group

Ahrens [17]	21/118 patients were not tolerant of the bed	NR
Bhazad [29]	NR	NR
Kirschenbaum [23]	6/17 developed pressure ulcers	5/20 developed pressure ulcers
Macintyre [7]	1/53 patients were intolerant of the bed	
	10% unplanned extubation	2% unplanned extubation
	2% loss of vascular lines	0% loss of vascular lines
	11% cardiac arrest	2% cardiac arrest
	23% new arrhythmia	14% new arrhythmia
	11% urinary tract infection	27% urinary tract infection
Gietzen [30]	NR	NR
Traver [27]	NR	NR
Whiteman [29]	5/33 patients were not tolerant of the bed	NR
	2/33 patients complained of neck or back stiffness	
deBloisbanc [19]	3/51 patients were not tolerant of the bed	NR
Nelson [24]	NR	NR
Shapiro [25]	4/16 patients were not tolerant of the bed	NR
Clemmer [18]	NR	NR
Fink [21]	13/51 patients taken off the kinetic therapy bed due to problems with skeletal traction, increased intracranial pressure, compression of unstable facial fractures or intolerance	NR
Demarest [20]	NR	NR
Summer [26]	1/41 facial ulcer	NR
	1/41 frequent premature ventricular contractions with turning	
Gentilello [22]	Decubitus ulcers 29.6%	Decubitus ulcers 26.3%

NR, not reported.
