# Supplementary figures and images for: Kinetic bed therapy to prevent nosocomial pneumonia in mechanically ventilated patients: a systematic review and meta-analysis
Source: Crit Care. 2006 May 9;10(3):R70. doi: 10.1186/cc4912 (PMC1550950; doi:10.1186/cc4912)

### Supplemental File 1:

Funnel Plot for the Effect of Kinetic Bed Therapy on Nosocomial Pneumonia

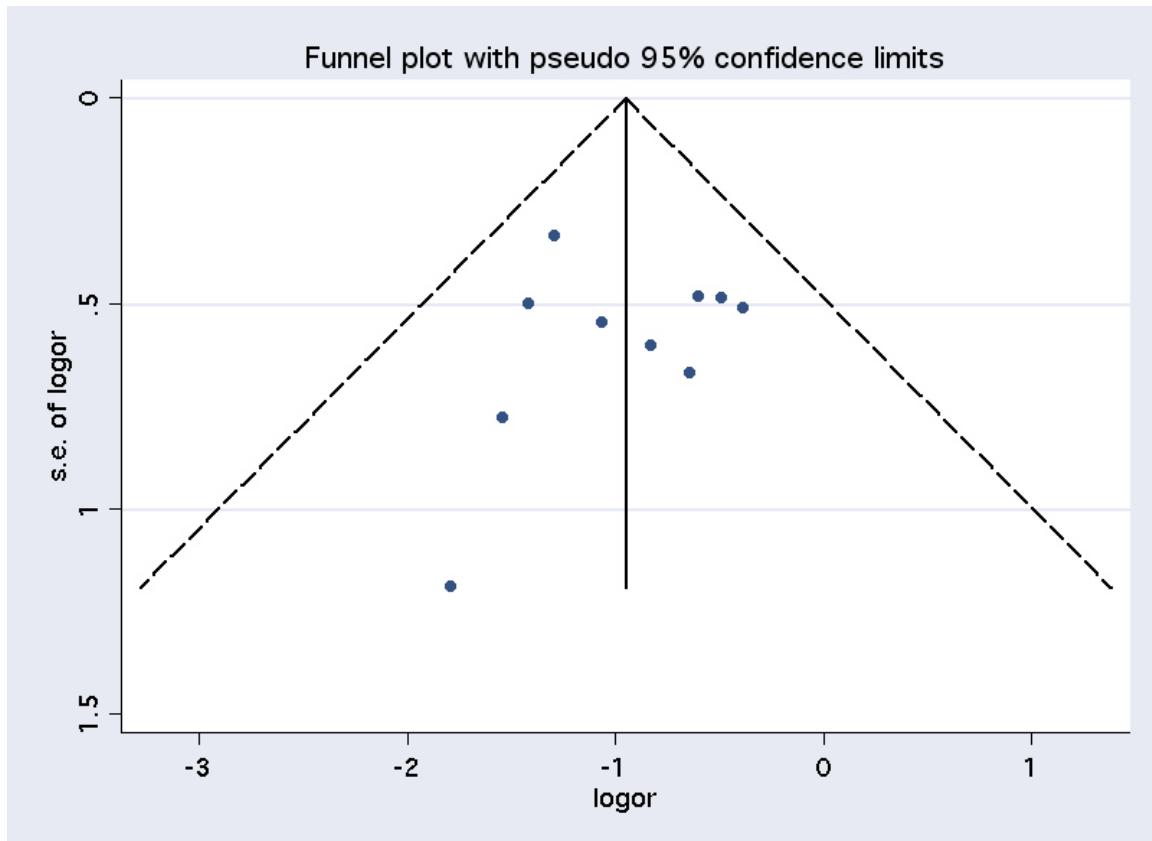

Supplement: Additional file 1 [file cc4912-S1.pdf]

### Supplemental File 5:

Funnel Plot for the Effect of Kinetic Bed Therapy on Mortality

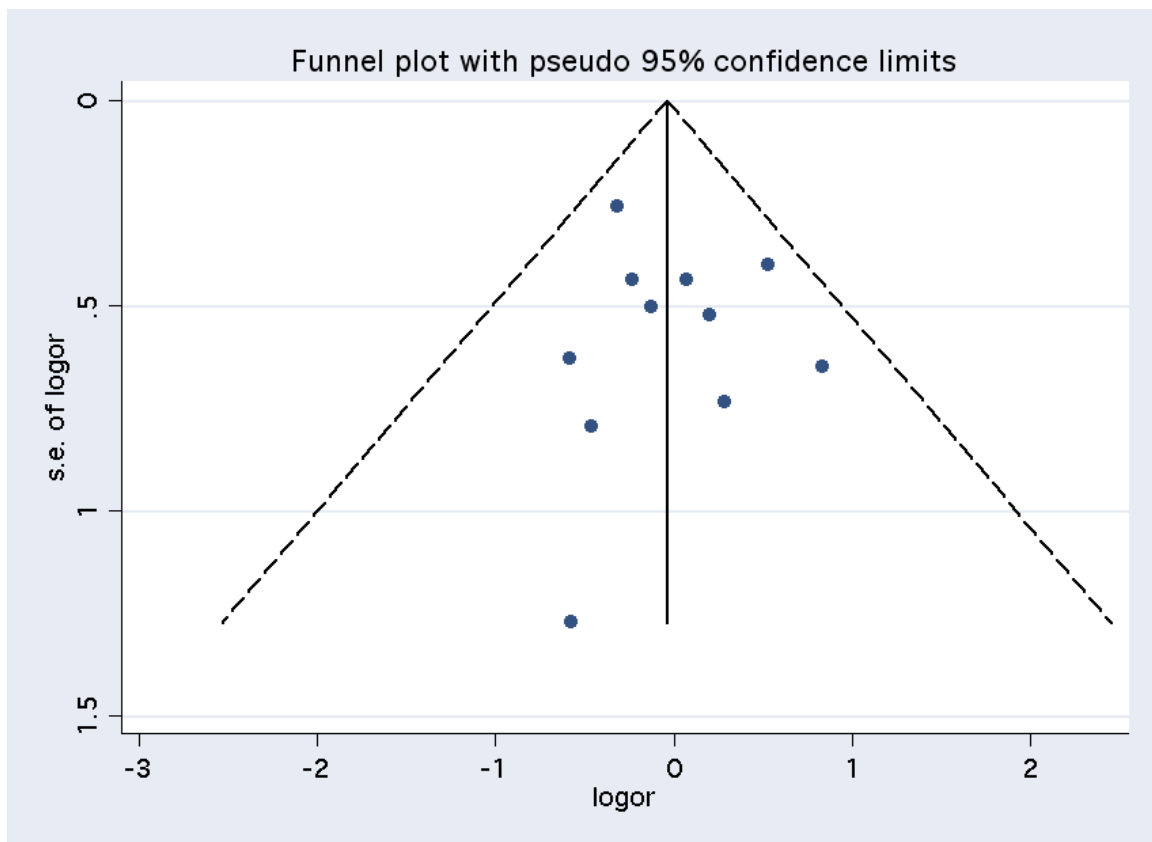

Supplement: Additional file 5 [file cc4912-S5.pdf]
