# Supplementary material for: Kinetic bed therapy to prevent nosocomial pneumonia in mechanically ventilated patients: a systematic review and meta-analysis
Source: Crit Care. 2006 May 9;10(3):R70. doi: 10.1186/cc4912 (PMC1550950; doi:10.1186/cc4912)

## Supplemental File 3:

Analysis Based on The presence or Absence of Blinding for the Effect of Kinetic Bed Therapy on the Incidence of Nosocomial Pneumonia

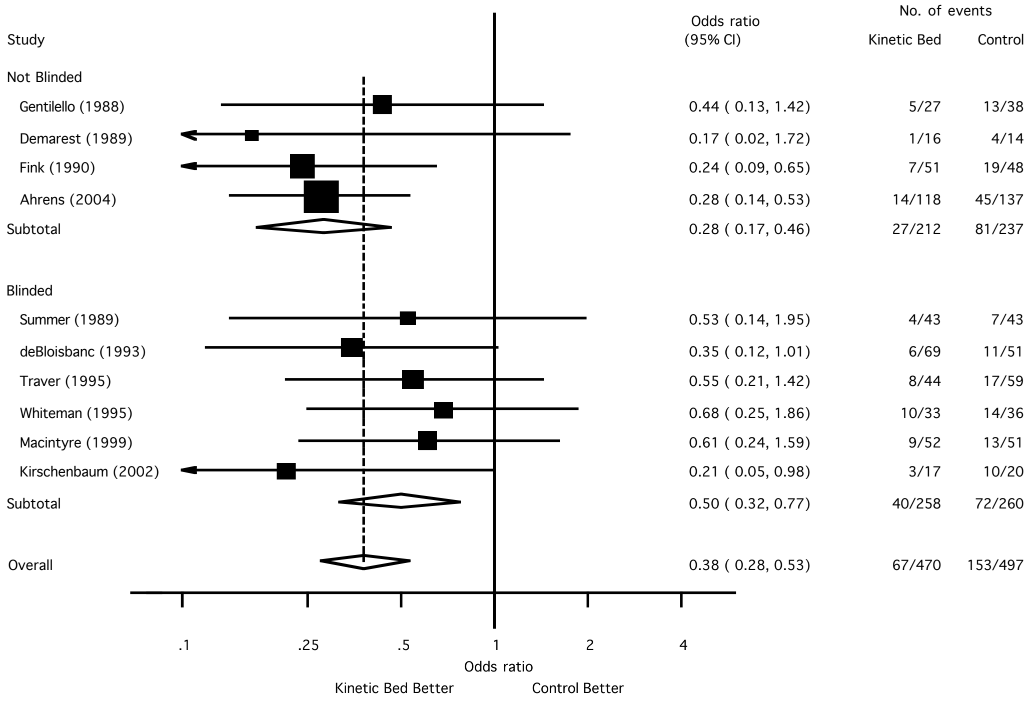

Supplement: Additional file 3 [file cc4912-S3.PDF]
