# Supplementary material for: Kinetic bed therapy to prevent nosocomial pneumonia in mechanically ventilated patients: a systematic review and meta-analysis
Source: Crit Care. 2006 May 9;10(3):R70. doi: 10.1186/cc4912 (PMC1550950; doi:10.1186/cc4912)
Supplement: Additional file 4 [file cc4912-S4.pdf]

Supplemental File 4:

Subgroup Analysis Based on the Arc of Rotation for the Effect of Kinetic Bed Therapy on the Incidence of Nosocomial Pneumonia

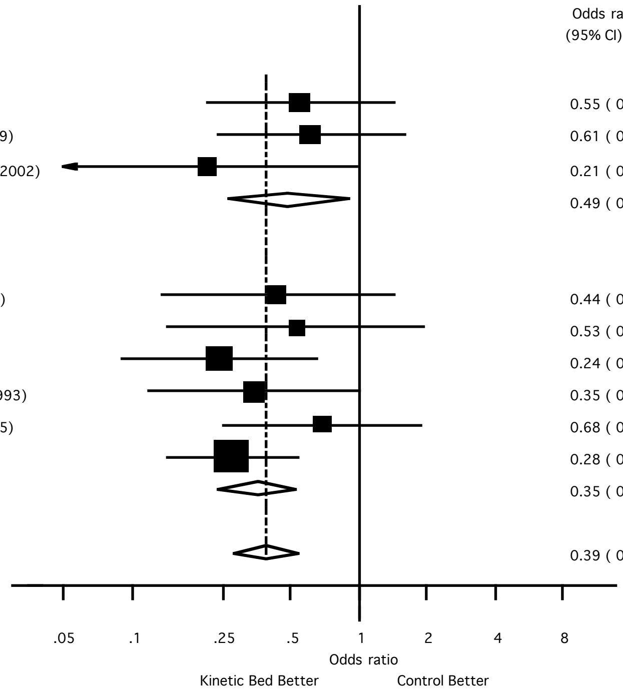

Study	Odds ratio (95% CI)	No. of events Kinetic Bed	No. of events Control
< 80 degrees			
Traver (1995)	0.55 (0.21, 1.42)	8/44	17/59
Macintyre (1999)	0.61 (0.24, 1.59)	9/52	13/51
Kirschenbaum (2002)	0.21 (0.05, 0.98)	3/17	10/20
Subtotal	0.49 (0.26, 0.90)	20/113	40/130
> 80 degrees			
Gentilello (1988)	0.44 (0.13, 1.42)	5/27	13/38
Summer (1989)	0.53 (0.14, 1.95)	4/43	7/43
Fink (1990)	0.24 (0.09, 0.65)	7/51	19/48
deBloisbanc (1993)	0.35 (0.12, 1.01)	6/69	11/51
Whiteman (1995)	0.68 (0.25, 1.86)	10/33	14/36
Ahrens (2004)	0.28 (0.14, 0.53)	14/118	45/137
Subtotal	0.35 (0.24, 0.52)	46/341	109/353
Overall	0.39 (0.28, 0.54)	66/454	149/483
