# Supplementary material for: Kinetic bed therapy to prevent nosocomial pneumonia in mechanically ventilated patients: a systematic review and meta-analysis
Source: Crit Care. 2006 May 9;10(3):R70. doi: 10.1186/cc4912 (PMC1550950; doi:10.1186/cc4912)

Supplemental File 6:

Analysis Based on the Studies With and Without Adequate Allocation Concealment for the Effect of Kinetic Bed Therapy on Mortality

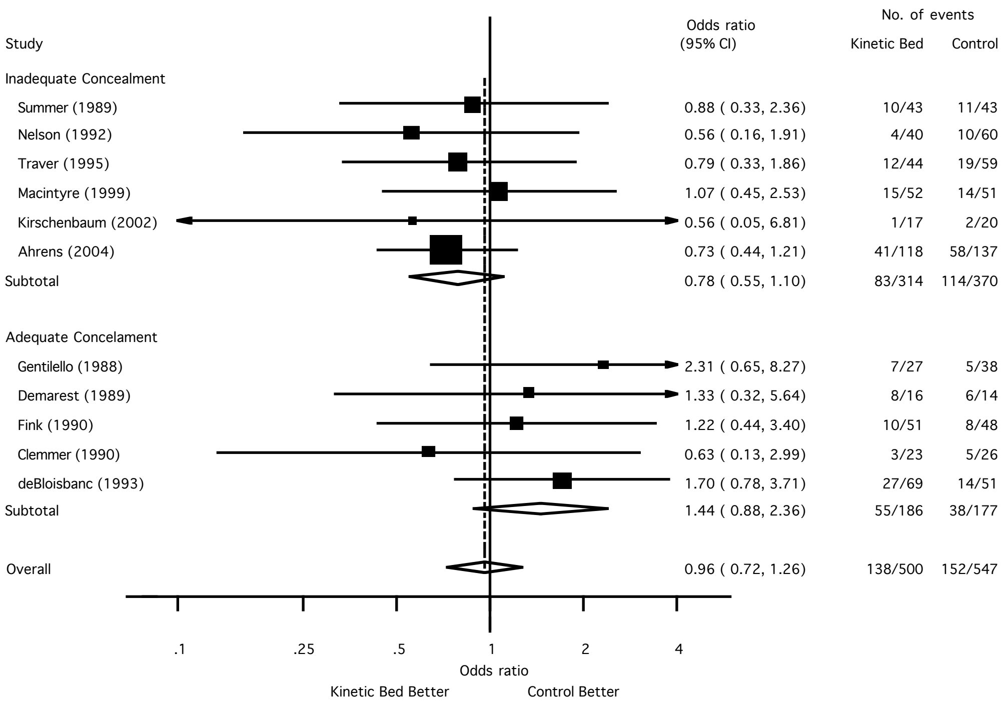

Supplement: Additional file 6 [file cc4912-S6.pdf]
